# Exploring the community phylogenetic structure along the slope aspect of subalpine meadows in the eastern Qinghai–Tibetan Plateau, China

**DOI:** 10.1002/ece3.5117

**Published:** 2019-04-01

**Authors:** Minxia Liu, Yingdi Che, Jiao Jiao, Lirong Li, Xiaoxuan Jiang

**Affiliations:** ^1^ College of Geography and Environment Science Northwest Normal University Lanzhou China

**Keywords:** community structure, environmental factors, phylogenetic, Qinghai–Tibetan Plateau, slope aspect

## Abstract

Exploring the community assembly has been important for explaining the maintenance mechanisms of biodiversity and species coexistence, in that it is a central issue in community ecology. Here, we examined patterns of the community phylogenetic structure of the subalpine meadow plant community along the slope gradient in the Qinghai–Tibetan Plateau of China. We surveyed all species and constructed the phylogenetic tree of the plant community based on data from the Angiosperm Phylogeny Group III. We selected the net relative index (NRI) and evaluated the community phylogenetic structure along the five slope plants communities. We found that the phylogenetic structure varied from phylogenetic clustering to phylogenetic overdispersion with the slope aspect from north to south. In the north slope, the community phylogenetically cluster indicated that the limiting similarity played a leading role in the community assembly and the maintenance of biodiversity. Community phylogenetic overdispersion in the east, southeast, and south slopes indicated that habitat filtration was the driving force for community assembly. The NRI index of the northeast slope was close to zero, implying random dispersion. But it may be driven by the neutral process or limiting similarity, in that the community assembly process was the result of a combination of several ecological factors and thus required further study.

## 1 INTRODUCTION

Community assembly research has been crucial to shed light on the coexistence of species and the maintenance of species diversity, in that it has always been the central topic of ecological research (Niu, Wang, Lian, Ye, & Shen, 2011; Niu, Liu, Shen, He, & Fang, 2009; Rosindell, Hubbell, & Etienne, 2011). The formation and maintenance of biological communities has been a long‐term evolutionary process, which was the result of the interaction between evolutionary and ecological processes (Niu et al., 2011; Zhang et al., 2016). The community assembly which was based on niche theory has dominated community ecology for nearly a century, yet understanding of the mechanisms of species coexistence has remained elusive (Niu et al., 2009).

Niche theory and neutral theory are two basic theories that attempt to explain the diversity of community species from different perspectives so as to reveal the community assembly mechanism (Hubbell, 2001). Niche theory held that every organism has the most suitable environment for survival to choose different balance strategies in different environments. The characteristics of organism reflected the adaptation to the environment. Niche theory predicted that the spatial pattern of a community should be closely related to environmental heterogeneity. The neutral theory assumed that species are niche equivalent, that species with the same niche can coexist, and that the number of species coexisting depends on the balance between species differentiation and random extinction. Ecological community assembly was the result of random drift and the ecological niche differentiation process, recognizing that niche and neutral processes do not have to diametrically oppose each other and a community is likely determined by the interplay of the two processes, and recently research attached great importance to the integrated ecological niche and neutral theory to explore the relative contribution of stochastic and deterministic effects to community assembly (Niu et al., 2009). Niche theory considered that habitat filtering and limiting similarity (competitive exclusion) were the two main process of community building and that they were jointly responsible for maintaining the stability of the community structure (Webb, Ackerly, Mcpeek, & Donoghue, 2002). Habitat filtering (Kraft et al., 2015) resulted in the similarity of characters, while limiting similarity (Godoy, Kraft, & Levine, 2014) led to divergence. Many studies have demonstrated the existence of environmental filtering and limiting similarity by constructing null model methods (Dante, Schamp, & Aarssen, 2013; Foster, Dickson, Murphy, KarelI, & Smith, 2004).

Many researches mainly explored the structure and diversity of the community from the environmental conditions and the ecological processes (predation, completion, mutualism, etc.) in the community (Goldberg & Miller, 1990). However, it has been neglected that historical factors have an important influence on the process of community assembly (Lu, Huang, Ci, Yang, & Li, 2014; Niu et al., 2011). Some scholars have proposed the application of phylogenetic biology to community ecology (Webb, 2000). Based on the phylogenetic structure of the species in the community, this method can be used to predict the influence of historical factors on the existing community and analyze the main causes of the community assembly (Webb et al., 2002). Because well‐resolved phylogenies that include all species of a study area are rare; botanists and plant ecologists have commonly used the super‐tree method to generate phylogenetic hypotheses for their phylogenetic studies of plant communities (Webb et al., 2002). Phylogenetic trees consisted of the node and evolutionary branch. Each of the nodes represented a taxonomic unit, and the evolutionary branch represented the taxa. The branch length was the result of the species’ evolution time. Mapping Phylogenetic trees, which are trees that are similar to branches of a tree, generalizes the genetic relationships of various organisms. Plant leaves were important bridges connecting plants to the external environment, and their functional character changes were influenced by the external environment and phylogeny (Sun et al., 2017). Different plant functional traits led to different community constructions (Gerhold et al., 2013). In the study of community system development, Webb divided ecological characters into conservative traits and convergence traits (Kraft, Cornwell, Webb, & Ackerly, 2007; Webb et al., 2002) (Table 1). Relationship between species should be considered in the correlation study on species traits to verify whether the species functioning traits show a phylogenetic signal. Previous community experiments on the subalpine meadow have shown that most function traits did not have a significant phylogenetic signal (Che, Liu, Li, Jiao, & Xiao, 2017; Gong & Wang, 2015; Liu et al., 2015). Studies have shown that not all functional traits show significant phylogenetic signals (Ding, Zang, Letcher, Liu, & He, 2012; Swenson, 2013). Compared with species of communities in the low‐altitude area, the species of the plateau environment were more threatened by the environment. To adapt to the conditions of drought and low temperature, species were more likely to undergo convergent evolution (Gong & Wang, 2015). Thus, we speculated that there was no obvious phylogenetic signal for the functional characters of the subalpine meadow in the slope gradient, and ecological traits were not involved in this study, which were assumed to be traits convergent.

**Table 1:** The excepted distribution of community phylogeny, given various community assembly processes and different evolutionary characteristics of ecological traits (after Webb et al., 2002; Kraft et al., 2007)

Community assembly processes	Evolutionary characteristic of ecological trait	
	Traits conserved	Traits convergent
Niche theory		
Habitat filtering	Cluster dispersion	Overdispersion
Limiting similarity	Overdispersion	Clustered or random dispersion
Neutral theory		
Neutral assembly	Random dispersion	Random dispersion

Ecological traits were not involved in our study, and traits were assumed to be convergent.

Currently, a series of studies have adopted the method of community phylogeny to explain the change in community composition better (Huang et al., 2014; Whitfeld, Kress, Erickson, & Weiblen, 2012). Whitfeld et al., (2012) found that environmental filtration was the main driving factor of community construction in the process of tropical forest succession in New Guinea. Yan et al. (2012) verified niche theory and neutral theory, respectively, on the basis of traits, indicating that similarity restriction can better determine the coexistence of species in subalpine forest community construction. Moreover, some of these studies (Bryant et al., 2008; Cao et al., 2013) have speculated on the driving factors of variability along the altitude base on community phylogeny. All the above studies confirm that there is some significant change in the community phylogenetic structure along the elevation (Lu et al., 2014).

As the youngest and highest plateau in the world, the Qinghai–Tibetan Plateau has been very sensitive to climate change (Liu et al., 2015; Xu et al., 2010; Xu & Xue, 2013; Zhang et al., 2012). There is a well‐known but barely acted‐upon issue that the ecosystem of the Qinghai–Tibetan Plateau is being destroyed by an extremely harsh natural environment and other damaging human activities. In recent decades, grassland degradation and biodiversity loss have been increasing, making the ecosystem very fragile. Once destroyed, it is difficult to recover, in that it is imperative to protect the alpine meadow grassland ecosystem (Zhang et al., 2016). Although a large number of investigations have been done on various aspects (species abundance distribution patterns; functional diversity; species diversity; stoichiometry characteristics; the influence of mowing and fertilization, grazing intensity on community) of the subalpine meadow community (Liu, Liu, Du, & Zhang, 2010; Wang, Zhang, & Zhu, 2013; Lü et al., 2014; Dong et al., 2017; Li et al., 2017), it was common to treat the different species of the phylogenetic status as the same, without regard to the relatedness of the species. Therefore, studying the community structure and the influence of environmental factors in the subalpine meadow has significant meaning.

This paper attempted to understand the nature of community development and to analyze the community structure of the subalpine meadow and the influence of environmental factors across different slope aspects to gain insights into community assembly. Therefore, we aimed to test whether the phylogenetic community structure had cluster dispersion, overdispersion or random dispersion in order to infer the dominant role of slope in community construction.

## 2 MATERIALS AND METHODS

### 2.1 Study site

We selected a study field in the Research Station of the Alpine Meadow and Wetland Ecosystem of Lanzhou University, which was situated at the Eastern part of Qinghai–Tibet Plateau, China (34°51′N, 102°53′E). The altitude was 2,900 m above sea level. The climate was humid‐alpine with a mean annual precipitation of 560 mm, most of which falls during summer. The mean annual temperature is 2.0°C. The coldest months (December–February) had a mean temperature of −10.6°C, and the warmest months (June–August) had an average temperature of 11.7°C. The area represents typical subalpine meadow. Its soil was classified as alpine meadow soil, and the plants were typical of species‐rich alpine meadows (Du & Wang, 1995), such as *Potentilla fruticose*, *Taraxacum mongolicum*, *Kobresia capillifolia*, and *Stipa capillata*.

### 2.2 Sampling design

**Figure 1:**
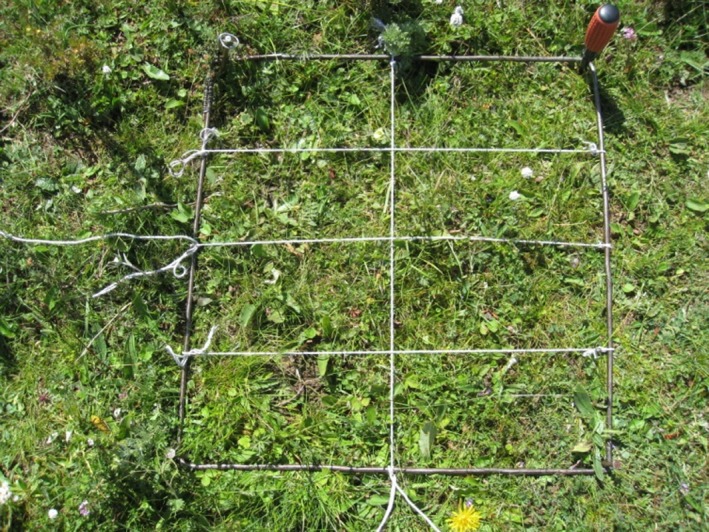
The figure of plots in study area

During the peak growing season of July in 2015, we conducted the surveys. We selected three hills, setting samples along the slope aspect (from north, northeast, east southeast, to south in sequence) (Figure 1). We counted the number of individuals for every species in each plot, measured the height of three randomly selected stems for each species, and estimated visually the cover of all species. We monitored the below‐ground soil temperature (10 cm deep below the soil surface, using an EM50 instrument, Shanghai), with three replicates of each measurement. Soil samples were collected from each plot at depths ranging from 0 to 20 cm below the soil surface.

### 2.3 Soil analysis

Soil samples were air‐dried and then passed through a 0.15‐mm sieve prior to analysis (with three replicates for each soil core). Soil water content (SWC) was measured as: SWC = (Mass_f_ − Mass_d_)/Mass_d_, where Mass_f _is the fresh mass of soil and Mass_d_ is the dry mass of soil. The content of soil organic carbon (SOC) was assayed by wet dichromate oxidation from a subsample of 0.2 g soil and titrated by FeSO_4_ using a titrator. Total soil organic nitrogen (STN) content was determined in air‐dried homogenized 0.5 g soil samples digested with sulfuric acid and a K_2_SO_4_:CuSO_4_:Se catalyst and analyzed using a SmartChem 200 discrete chemistry analyser. Total soil organic phosphate (STP) content was determined by digesting with H_2_SO_4_‐HClO_4_ and analyzed using the Olsen method. We measured concentrations of available nitrogen (SAN) and available phosphorus (SAP) with a SmartChem Discrete Auto Analyser. Soil pH was determined using a 2.5:1 water to air‐dried soil ratio and a standard pH meter.

### 2.4 Functional traits

Choosing eight functional traits, it was generally considered that these were significant for plants and directly related to plant interactions: leaf nitrogen, phosphorus and potassium content (mg/g), leaf organic carbon content (mg/g), specific leaf area (cm^2^/g), leaf dry matter content (mg/g), leaf water content (%), and chlorophyll content. We used a scanner to scan the mature and undamaged leaves of five randomly selected individuals per species and then saved these pictures and used ImageJ to estimate the leaf area. We weighed the fresh weight of the fresh leaves and dried leaves. Then, we tested the leaf dry matter content (LDMC) and specific leaf area (SLA) of each species on each slope aspect. SLA*_i_* = leaf area/leaf dry mass, where leaf area is the selected total area of species *i *and the leaf dry mass is the total dry mass. LDMC* = *leaf dry mass/leaf fresh mass. We measured the leaf dry mass and nitrogen and phosphorus content (expressed as mg/g) from leaves collected from the same individuals. The total nitrogen (N) of plants was determined by the micro‐Kjeldahl method of means of micronutrients. The plant total phosphorus (P) determination of molybdenum antimony colorimetry was obtained by using H_2_SO_4_‐H_2_O_2_. The total potassium (K) of the plant was determined by the flame photometric method (H_2_SO_4_‐H_2_O_2_). The plant organic carbon was determined by the potassium dichromate capacity method. The SPAD value of the plant leaves was measured with a portable chlorophyll instrument (SPAD‐502, Minolta Camera Co., Osaka, Japan).

To better reveal the interaction relationship between the small terrain environment factor and plant leaf characteristics in subalpine meadow communities, this study selected an eight‐plant functional traits index as the species and selected eight soil factors as the environment and then used the two groups of variables in a redundancy analysis (RDA).

### 2.5 Phylogeny

The community phylogeny was developed mainly through the community phylogenetic relationship index (net relatedness index, NRI; nearest taxon index, NTI) to detect the existence of a phylogenetic structure of the community. This paper chose the net relatedness index (NRI measures the average lineage distance between community species and focuses on the overall description of the lineages of species in the community), and the NRI index is based on the overall level within community and reflects the development pattern of the entire phylogenetic tree (Kraft et al., 2007). We selected Webb's (2000) method to estimate phylogenetic community structure and constructed a super‐tree representing the species pool. We briefly described the phylogenetic methods here. NEXUS is one of the standard file formats for phylomatic. For the 88 species recorded in our study, all species were classified according to Angiosperm Phylogeny Group III (APG III). Furthermore, because the super‐tree did not include information about branch length, it must use the branch length adjustment function (BLADJ) implemented in Phylocom (Webb, 2000; Webb et al., 2002). The software returned a new phylogeny with adjusted branch lengths and calculated Faith's index of phylogenetic diversity (PD) to quantify phylogenetic distance of pairwise species (Faith, 1992).

To measure the phylogenetic community structure, we always chose the method of comparison with the null model. Choosing an appropriate null model requires careful consideration. Every null model makes different assumptions, and using two null models to analyze the same data can give radically different results. We chose the null model 2 in this paper. In null model 2, species from each sample become random draws from the phylogeny pool. Furthermore, this null model maintains the species richness of each sample, but the identities of the species occurring in each sample are randomized.

Assuming that the species distribution was random, the genealogical distance is standardized under the stochastic model to obtain the community system development structure index, namely the net affinity index (NRI).

$${\text{NRI}}_{s}=-1\times \frac{{\text{MPD}}_{s}-{\text{MPD}}_{\mathrm{rnds}}}{\text{SD}({\text{MPD}}_{\mathrm{rnds}})},$$

where MPD_s_ was the mean pairwise distance of samples and MPD_rnds_ was the mean pairwise distance of samples under the null model 2 (the MPD for each subplot was computed using the “picante” package in R version 3.2.4). If the value of NRI_s _was greater than that of the null model, the sample's phylogenetic community structure was cluster dispersion; if the value of NRI_s _was less than that of the null model, the sample's phylogenetic community structure was overdispersion; and if the value of NRI_s _was equal to that of the null model, the sample's phylogenetic community structure was random dispersion.

## 3 RESULTS

### 3.1 RDA of plant leaf characteristics and environmental factors by the slope aspect

**Figure 2:**
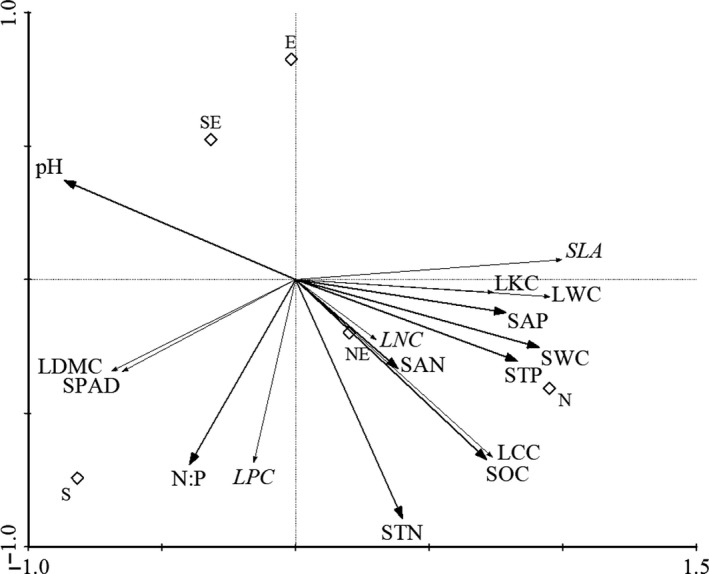
RDA two‐dimensional ordination diagram of plant leaf characteristics and environmental factors. N: north slope; EN: northeast slope; E: east slope; SE: southeast slope; S: south slope; SWC: soil water content; SOC: soil organic carbon; SAN: soil available nitrogen; SAP: soil available phosphorus; STP: soil total phosphorus; STN: soil total phosphorus; N:P: soil total nitrogen: soil total phosphorus; pH: soil acidity and alkalinity; SLA: specific leaf area; LDMC: leaf dry matter content; LWC: leaf water content; LCC: leaf carbon content; LKC: leaf kalium content; LPC: leaf phosphoru content; LNC: leaf nitrogen content; SPAD: chlorophyll content.

As the RDA figure (Figure 2) shows, the change of slope direction has a significant effect on soil water content, soil nutrients (carbon, nitrogen, phosphorus, and N:P), and soil acidity and alkalinity (pH), etc. Plant function traits LWC, LKC, SLA, LNC, and LCC with soil factors STP, SWC, STN, SAN, SOC, and SAP had a positive correlation, and the degree of correlation was LWC > SLA > LKC > LNC > LCC. Plant function traits LDMC, SPAD, and LPC with soil factors pH and N:P had a positive correlation. Moreover, most of the plant leaf functional traits had a significant difference along the five slope aspects. The SWC and LWC were highest in the north slope, while LDMC and SPAD were highest in the south slope. Thus, the habit of north slope was surely suitable for plant growth, and the highest leaf moisture content indicated the thickest leaf in north slope.

Table 2 displays the correlation between the environmental factors and the sorting axes of the five slope aspects. The first sorting axis had significantly positive correlation with SWC and STP, which had a significantly negative correlation with pH (*p* < 0.01). The first sorting axis was positive correlation with SOC and SAP (*p* < 0.05). Sorting shaft 1 contains the most information about the environmental factors as can be seen in the RDA sort figure (Figure 2). The first sorting axis mainly reflected the change of SWC, STP, SOC, SAP, and pH along the slope aspect. The information contained in the second axis of different slopes was more complex. The largest correlation coefficients of the second axis were STN (−0.8936), N:P (−0.6914), and SOC (−0.6735). Environmental factors for plant leaf characteristics in the first and second axes of accumulation were accounted for by the sum of eigenvalues as more than 95%. Table 2 indicated that the eigenvalues of the first and second axes were 88.4% and 7.2%, respectively. The result shows that the sorting axis reflects most of the information between environmental factors and leaf traits.

**Table 2:** RDA analysis of ordination axes and soil factors

Soil factors	SPEC AX1	SPEC AX2	SPEC AX3	SPEC AX4
SWC	0.9113**	−0.2539	−0.2357	0.2225
STN	0.4002	−0.8936**	0.0940	−0.1801
STP	0.8294**	−0.3044	−0.2691	0.3834
*N*:P	−0.3970	−0.6914*	0.3635	−0.4819
SOC	0.7145*	−0.6735*	−0.0536	0.1817
SAN	0.3827	−0.3321	−0.6120	−0.6072
SAP	0.7873*	−0.1237	0.6040	−0.0088
pH	−0.8656**	0.3702	0.2086	−0.2650
Eigenvalues	0.884	0.072	0.034	0.010

* Significant correlation at *p* < 0.05 level.

** Significant correlation at *p* < 0.01 level.

### 3.2 Community phylogenetic tree and variation of phylogenetic diversity

**Figure 3:**
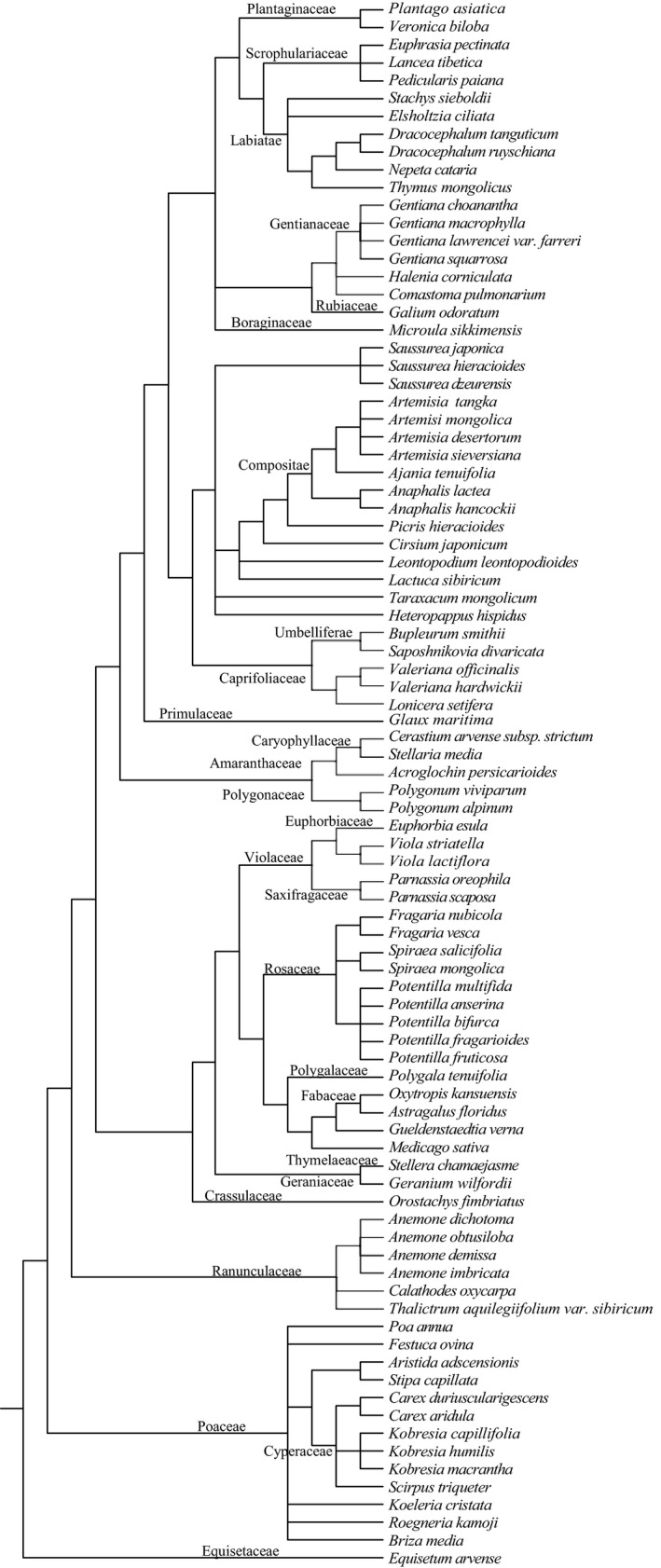
A super‐tree representing the 88 species based on slope‐oriented plots in subalpine meadow communities of subalpine meadows in the eastern Qinghai–Tibetan Plateau, China

A super‐tree represents the 88 species based on slope‐oriented plots in subalpine meadow communities of subalpine meadows in the eastern Qinghai–Tibetan Plateau. As seen in Figure 3, species belonging to the same family were gathered together. Phylogenetic tree was also called molecular evolutionary tree that consisted of the node and evolutionary branch. Each of the nodes represented a taxonomic unit (individual species, etc.), and the evolutionary branch represented the taxa (ancestors and descendants). The relationship between one branch could connect the two adjacent nodes, and branch length was the result of the species’ evolution time. For example, *Potentilla multifida*, *Potentilla anserina*, *Potentilla bifurca*, *Potentilla fragarioides*, and *Potentilla fruticosa* were belonged to Potentilla multifida, with the same node and branch length, indicating the same evolutionary time. So did the *Fragaria vesca* and *Fragaria nubicola*.

As Table 3 shows, the phylogenetic diversity index was reduced with changes in the slope aspect from north to south. The numbers of species had a similar variation trend along the slope aspect. The total branch length connected the two species by using the PD function in the *picante *package (Kembel et al., 2010). Thus, PD is inversely proportional to species relatedness, with species becoming less related as PD increases.

**Table 3:** Variation of phylogenetic diversity along slope aspect

Slope aspect	ntaxa	PD	tree Branch Length	propTree Branch Length
North slope	51	108	149	0.725
Northeast slope	41	88	149	0.591
East slope	34	84	149	0.564
Southeast slope	29	79	149	0.530
South slope	23	71	149	0.477

### 3.3 PCA ordination diagram of species along the five slope aspects

**Figure 4:**
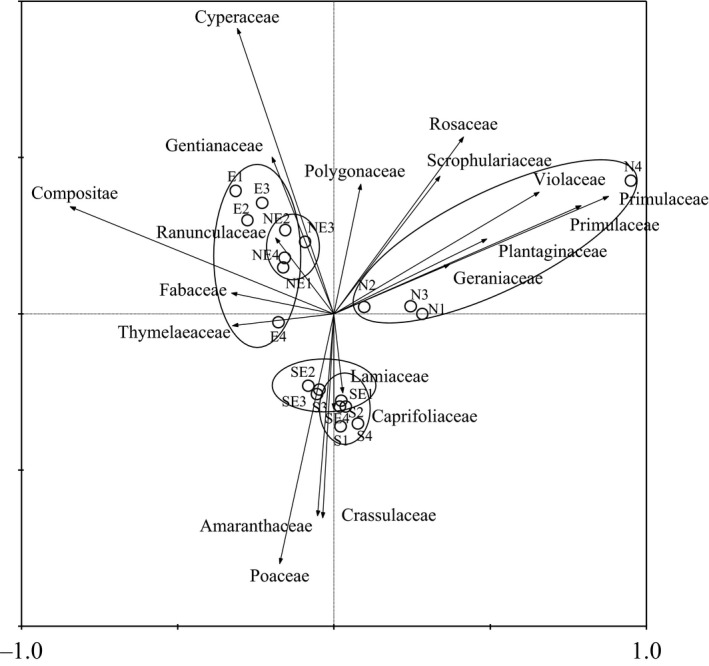
Two‐dimensional PCA ordination diagram of twenty quadrats c ommunities in the five slope aspects. N1: north slope quadrat 1; N2: north slope quadrat 2; N3: north slope quadrat 3; N4: north slope quadrat 4; NE1: northeast slope quadrat 1; NE2: northeast slope quadrat 2; NE3: northeast slope quadrat 3; NE4: northeast slope quadrat 4; E1: east slope quadrat 1; E2: east slope quadrat 2; E3: east slope quadrat 3; E4: east slope quadrat 4; SE1: southeast slope quadrat 1; SE2: southeast slope quadrat 2; SE3: southeast slope quadrat 3; SE4: southeast slope quadrat 4; S1: south slope quadrat 1; S2: south slope quadrat 2; S3: south slope quadrat 3; S4: south slope quadrat 4.

For the simplicity of the sequence diagram (Figure 4), we omitted those families whose fitness value was less than 10% and those species with a small explain contribution to the sorting chart. The interpretation of Figure 4 is threefold. First, as Figure 4 shows, each small circle represents a quadrat, big ellipse represents a slope community sample, and the relationship between the quadrats can be directly expressed by the length of the connection between quadrat s. The shorter the attachment is, the smaller the differences between samples. The distance between the south slope and the south slope community samples was close. Therefore, the difference between the two communities was also small. The anisotropy of the other samples was significant, and there were four distinct sampling areas in the sequence diagram. Moreover, we can observe the distribution of families. As seen from Figure 4, the sample communities were dominated by Primulaceae and Rosaceae in the north slope aspect, such as *Potentilla multifida*, *Potentilla anserina*, *Potentilla bifurca*, *Potentilla fragarioides*, and *Potentilla fruticosa*. The northeast slope was dominated by Cyperaceae, and the east slope was dominated by Compositae. The southeast slope and the south slope were dominated by Poaceae. Third, Figure 4 also reveals the number of the plants along the slope aspect. The species was mostly grown in the north slope, followed by the northeast and east slopes, while it was least grown in the southeast and south slopes. This result also indicated that the habit of north slope was surely suitable for plant growth.

**Table 4:** The eigenvalue of four axes of PCA analysis of quadrats information

Axes	1	2	3	4	Total variance
Eigenvalues	0.440	0.291	0.128	0.064	1.000
Cumulative percentage variance of species data	44.0	73.1	85.9	92.2	
Sum of all eigenvalues					1.000

Principal component analysis reveals the main direction of things through a linear combination of the original variables (i.e., the principal component). In this analysis, the eigenvalues of the four axes were 0.440, 0.291, 0.128, and 0.064, and the cumulative interpretation of the four components to the sample data was 92.2% (Table 4). The first and second component axis explained that most of the species variables were 44.0% and 29.1%, respectively. Therefore, the first and second component axis can be used as the principal component axis.

### 3.4 Variation of community phylogenetic relatedness along the slope aspect

**Figure 5:**
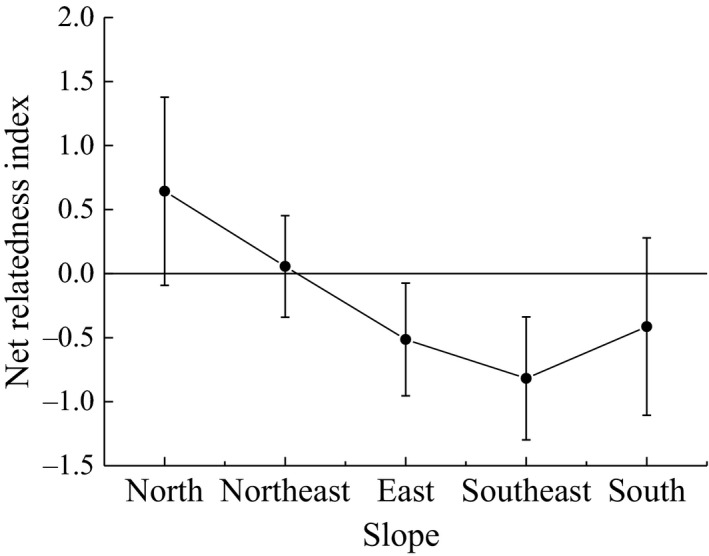
Variation of community phylogenetic relatedness along the slope aspect (mean ± *SD*). Different lowercase letters indicate significant difference among different slope aspects (*p* < 0.05)

The community phylogenetic relatedness NRI decreased with changes in the slope aspect from north to southeast. As Figure 5 shows, the value of the NRI was more than zero in the north slope, while in the east, southeast, and south slopes, the values were less than zero. Thus, the sample's phylogenetic community structure of the north was cluster dispersion. Moreover, in the east, southeast, and south slopes, the sample's phylogenetic community structure was overdispersion. However, the value of the NRI over northeast slope was close to zero. So the northeast slope of phylogenetic structure may be random, but this was not necessarily caused by neutral role. Other ecological processes will also affect the pattern of species diversity, such as predation among organisms.

## 4 DISCUSSION

Community assembly is a topic of considerable importance. Understanding the maintenance mechanisms of the species, coexistence, and biodiversity is not only essential for community assembly but also for the prediction of how plant communities respond to environmental change (Chai & Yue, 2016).

### 4.1 Effects of environmental factors on species leaf functional traits

Soil, an important source of plant nutrition, its distribution and change had an effect on the growth and development of plants (Liu, Che, Li, Jiao, & Xiao, 2017). The results of the RDA analysis showed the relationship between the characteristics of plant leaf characters and environmental factors. The differences in external conditions (soil water content, soil nutrients, etc.) due to the difference in terrain affected the composition of the plant community structure and the changes in plant leaf characters. The soil moisture content was the highest in the north slope because the degree of illumination in the north slope was weak, the light time was short, and the water evaporation was small. Soil nutrients were higher in the north and northeast slopes. This finding was consistent with the findings of Gong et al. (2008). The results of RDA analysis show the relationship between the character of the plant leaf and environmental factors. The soil moisture content, soil total phosphorus content, and pH are the main control factors (Figure 2, Table 2). This result was consistent with the research results of Liu (2017a, 2017b). The results showed that the soil moisture content was significantly correlated with the relative water content of plant leaves, leaf nitrogen, potassium content, leaf organic carbon content, and specific leaf area. Relevant studies also suggested that the difference in the distribution of soil moisture content caused by the slope is the most important factor affecting plant leaf characteristics in the habitat (Li, 2011; Liu et al., 2017; Ma et al., 2008). Therefore, we can conclude that illumination and soil water content were the primary environmental limiting factors affecting the habitat of the subalpine meadow.

### 4.2 The variation of phylogenetic diversity and community diversity

The results of this study showed that the species diversity index and phylogenetic diversity had the same change trend (Table 3) from the north to the south slope, and the number of plants and the system phylogenetic index were decreased. This finding was the same as that in a previous study by Honorio Coronado et al. (2015). The possible explanation for this conclusion was that the phylogenetic diversity index was the sum of the length of the phylogeny branches (Figure 3, phylogenetic tree, which revealed the family of species and the species’ evolution time) of the taxon in a certain sample (Faith, 1992; Kembel et al., 2010). The value of the phylogenetic diversity index indicates that the species’ phylogenetic diversity in the sample was the proportion of phylogenetic diversity in the whole region (Faith, 1992; Kraft et al., 2007). Figure 4 shows the principal component of the sample communities in the five slope aspects: the sample communities were dominated by Primulaceae and Rosaceae, Cyperaceae, Compositae, and Poaceae from the north to south slope aspect. The sample communities were dominated by Primulaceae and Rosaceae in the north slope aspect. Because the north slope quadrat 4 had many *Glaux maritima* of Primulaceae, and the thicket, *Potentilla fruticose*, inhibited the growth of the Poaceae and Leguminosae (Liu, 2017b). Figure 4 also reveals the distribution of the species along the slope aspect. The species distribution showed regular changes in the slope, and the north slope species were the most distributed, followed by those of the northeast and east slopes, while those of the southeast and south slope were less distributed. This result is consistent with the research results of Nie, Li, and Wang (2010), which showed that the diversity of species is negatively correlated with light and soil temperature and is positively correlated with soil moisture content. This correlation was because the number of species was related to its habitat: the soil moisture content and the soil nutrient content in the north and northeast slopes were higher, which is suitable for the survival of most plants and provides a suitable living environment for shade‐tolerant species, increasing the heterogeneity of the north slope and providing favorable conditions for the survival of rare species (Callaway et al., 2002; Gong & Wang, 2015; Liu, 2017b). Thus, there was a positive correlation between phylogenetic diversity and species abundance.

### 4.3 The community construction process and driving factors of subalpine meadow

The subalpine meadow plant community phylogenetic structure decreased gradually with the change in the slope aspect from north to south (Figure 5), and previous investigations have obtained the same conclusion (Che et al., 2017; Gong & Wang, 2015). Combined with environmental factors, functional traits, and phylogeny, we can observe the following: the functional characters of plant leaves showed regular changes in the slope direction, and the phylogenetic structure decreased gradually with the change in slope aspect (Figures 2, 3, 4, 5). In the north slopes, the community phylogenetic structures were cluster dispersion, which revealed that limiting similarity was the main driving force of community assembly. The main reason for the difference in the environmental pattern of the north and south slopes is that the difference of solar radiation drives the change in hydrothermal factors in the habitat gradient along the slope (Liu, 2017b; Nie et al., 2010). Because north slopes had the higher soil moisture and soil nutrients which are suitable for the survival of most plants (Li, 2011; Sternberg & Shoshany, 2001) and the higher moisture content and the lowest dry matter content in the leaves of plants in the north slope, all this indicated that the plant leaves have better growth conditions (Figure 2). Thus, the biological interaction between organisms became increasingly enhanced. In addition, the north slope of dense thickets for other plants provided a shade environment and occupied a large living space (Figure 4) (Gong & Wang, 2015). Thus, the community assembly mechanism in the north slope may be driven by the interaction between organisms (Table 1), which includes both the exclusion of competition and the positive interaction between organisms. Wang et al. (2014) held a phase of the alpine meadow plant with indirect positive interaction; their research results showed that the *Potentilla fruticosa* effect on the performance of other species is different, and there are indirect positive interactions and additional competition. The NRI index of the northeast was close to the null model (Figure 5), and there was neither significant cluster dispersion nor significant overdispersion (Table 1). It is not easy to think that the community assembly in the northeast was driven by the neutral process because this situation may also be caused by the combination of habitat filtering and competition exclusion (Mayfield & Levine, 2010). Accumulated empirical evidence shows that both deterministic and stochastic mechanisms influence the processes of plant community assembly (Chase, 2003; Chave, 2004; Fukami, Bezemer, Mortimer, & Putten, 2005; Kraft & Ackerly, 2010; Kraft, Valencia, & Ackerly, 2008; Sterck, Markesteijn, Schieving, & Poorter, 2011). From a phylogenetic perspective, the community structure of the east, southeast, and southern slope plants was phylogenetic overdispersion (Figure 5). Therefore, the driving force of community assembly was habitat filtering. From the perspective of the functional traits, the soil moisture and soil nutrient content were low, while illumination was strong (Li, 2011; Sternberg & Shoshany, 2001). Therefore, the living environment is adverse for plant growth, and there are few species (Figures 2 and 4). This result showed that the conditions for the growth of species are greatly influenced by the environment, as described by Xiao and Wang (2015).

## 5 CONCLUSION

In our study of the subalpine meadows in the eastern Qinghai–Tibetan Plateau, China, we used both environmental factors and functional trait to investigate the mechanisms of assembly and found strong support for the role of deterministic biotic interactions. We can conclude that illumination and soil water content were the primary environmental limiting factors affecting the habitat of the subalpine meadow. There was a positive correlation between phylogenetic diversity and species number. The number of species was related to its habitat. Habitat filtering was the main assembly mechanism of the east, southeast, and south slope plant communities, and the limiting similarity was the dominant force in the north slopes. It is not easy to think that the community assembly in the northeast was driven by the neutral process because this situation may also be caused by a combination of habitat filtering and competition exclusion. These findings imply that the processes that govern species diversity in this landscape operate at multiple scales, an understanding that is important for conservation and restoration of meadow plant communities.

## CONFLICT OF INTEREST

None declared.

## AUTHORS’ CONTRIBUTIONS

Minxia Liu, Yingdi Che designed research; Minxia Liu, Yingdi Che, Jiao Jiao, Lirong Li, Xiaoxuan Jiang participated in field experiments and laboratory experiments; Yingdi Che analyzed data and wrote the paper; Minxia Liu and Yingdi Che revised the paper.

## Data Availability

Species family data based on Angiosperm Phylogeny Group (APG) III and http://frps.eflora.cn/.
